# Geographic Mapping of Crohn's Disease and Its Relation to Affluence in Jiangsu Province, an Eastern Coastal Province of China

**DOI:** 10.1155/2014/590467

**Published:** 2014-04-15

**Authors:** Dong Hu, Jianan Ren, Gefei Wang, Guosheng Gu, Song Liu, Xiuwen Wu, Jun Chen, Huajian Ren, Zhiwu Hong, Jieshou Li

**Affiliations:** Department of Surgery, Jinling Hospital, Medical School of Nanjing University, 305 East Zhongshan Road, Nanjing 210002, China

## Abstract

*Background*. Geographical variation in the incidence of Crohn's disease (CD) has been reported in Europe and North American. However, there are no comparable data in mainland China. *Methods*. We retrospectively identified incident cases of CD patients registered in Jinling hospital during 2003 to 2012. The standardized incidence ratio (SIR) was calculated for each area of Jiangsu province and a thematic map of CD was made according to the local SIR. The association between incidence and local economic status was revealed by correlation between SIR of CD and different local economic indicators. *Results*. A total of 653 CD patients (male-to-female ratio, 1.8 : 1) from Jiangsu province were included. A steady increase was observed in the number of CD patients over the period of observation. Disease map of SIR showed a pronounced geographic concentration of CD in the south part of Jiangsu province. Spearman correlation analysis showed a positive correlation between local SIR of CD and local economic indicators. *Conclusions*. There is a marked geographic variability in CD incidence across Jiangsu province. CD incidence in affluent areas seems to be higher than that in less affluent areas. Further multicenter population-based studies are needed to assess the real disease map of CD.

## 1. Introduction

The incidence rates of Crohn's disease (CD) markedly differ geographically and among different ethnic groups presumably due to genetic and environmental factors [1–3]. Epidemiological studies show that the global map of CD can be broken down into several geographic zones according to the variation of incidence [3, 4]. It seems that CD is a disease of the western world [4]. However, a rising incidence and prevalence of CD has been recently observed in countries with traditionally low rates of CD, especially in Asian countries including China [5, 6].

China is a developing country which holds the largest population in the world [7]. The economic growth in China was visible since the Reform and Open Policy in 1978, followed by the increasing gap between the poor and the rich [8, 9]. On the other hand, compared to 1990, the nationwide ratio of patients with CD to total hospitalized patients has increased by 2.78 times in 2003 [10]. Also a study from Scotland showed that children from more affluent areas had a higher relative risk of developing CD [11]. As a result, the correlation between surging economy and the increasing incidence of CD in China has attracted a great deal of attention.

However, to our best knowledge, no study has been carried out to investigate the CD incidence and its relation to affluence in mainland China. Thus, we carried out a retrospective study based on the database of a large tertiary hospital, to investigate the geographic variation of CD in Jiangsu province, an eastern coastal province of China, and its relation to local economic status.

## 2. Materials and Methods

### 2.1. Study Setting and Patients

Jiangsu province is situated at the center of the eastern coast of China, with a population of approximately 79.2 million. It covers a total area of 102,600 sq. km, making up 1.06% of China's total territory.

Jinling Hospital is a large tertiary hospital in Jiangsu province. Each year patients with CD from all over mainland China come to Jinling Hospital for further treatment. We selected patients through searching for “Crohn's disease,” “CD,” “inflammatory bowel disease,” “colitis,” “IBD,” or “enteritis” among discharge diagnosis of patient's medical report. Medical records of CD patients who registered in Jinling Hospital during the period of 2003 to 2012 were reviewed retrospectively. We also checked with endoscopy lists, pathology reports, and IBD team records, which linked with medical records system in our hospital. Two senior physicians (GW and GS) confirmed the diagnosis of CD by review of the case records and reference to the diagnostic criteria [12].

In our hospital, the diagnosis of adult CD should be based on the combination of physical examination, colonoscopy (with multiple biopsies), laboratory investigations (erythrocyte sedimentation rate, C reactive protein, and calprotectin), and small bowel imaging (computed tomography enterography (CTE) or magnetic resonance enterography (MRE)). For pediatric CD patients, initial investigation should also include double-balloon enteroscopy and assessment of nutritional condition and growth level. All patients involved in current study are hospitalized patients who have received or confirmed their CD's diagnosis in Jinling Hospital.

### 2.2. Data Sources

The main data collected include demographics, city of residence at diagnosis, and clinical parameters (including the disease classification [13, 14], radiological, endoscopic, and histological findings at the time of diagnosis). Here we choose several economic indicators to measure the local economic status, including saving deposits per capita, annual income per capita, disposable expenditure per capita, living expenditure per capita, gross domestic product (GDP) per capita, and Engel coefficient. The Engel coefficient is the proportion of family income that is spent on food. It is well accepted that the percentage of income families spent on food declined as their income level rose [15]. All the economic data mentioned in current study came from the annual reports published on the website of China's National Bureau of Statistics (CNBS, http://www.stats.gov.cn/english/).

### 2.3. Disease Map

The incidence rates of CD were calculated by the number of registered CD cases during the period of 2003 to 2013 divided by the population at risk (population of different areas of Jiangsu province). Standardized incidence ratios (SIRs) were estimated for each of the 13 areas of Jiangsu province, using regional gender- and age-specific rates as a reference. SIR is the ratio of the number of cases actually observed to the number expected. The latter was calculated by applying the age-specific incidence rate of the whole population to the number of population in each province. Gradient colors were applied into different areas in the map of Jiangsu province according to its SIR to show the distribution of patients intuitively.

### 2.4. Statistical Analysis

Statistical analysis was performed using GraphPad Prism Software (version 5.01; GraphPad, San Diego, CA). Data visualization was performed using PowerPoint software (version 2010, Microsoft). All analyses were two-tailed and differences were considered statistically significant when *P* value < 0.05. For continuous variables, mean and standard error of mean (SEM) were calculated. Student's *t*-test was used to compare variance between groups. For categorical variables, percentages were provided and chi-squared test was used. Spearman analysis was used to calculate the correlation between number of CD patients and local economic indicators.

## 3. Results

### 3.1. General Results

A total of 1446 cases were identified with CD when searching for “Crohn's disease,” “CD,” “inflammatory bowel disease,” “colitis,” “IBD,” or “enteritis” among discharge diagnosis of medical reports. Fifty-four cases were excluded (33 patients were excluded due to lack of data and 21 patients were identified to be misdiagnosed with CD after surgery or during their second/third time of hospitalization).

**Figure 1:**
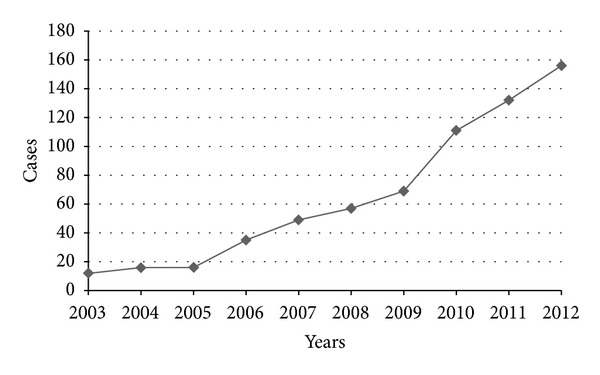
Annual newly registered CD patients from Jiangsu province between 2003 and 2012.

**Figure 2:**
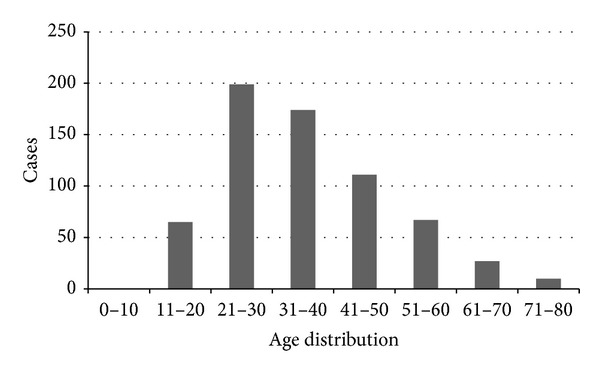
Age distribution of CD patients when registration.

Among the rest 1392 CD patients, 653 (420 male and 233 female; male-to-female ratio is 1.8 : 1) came from Jiangsu province. There are 26 pediatric patients (below 17 years old) and 627 adult patients (above 17 years old). All patients were ethnically Chinese. A steady increase was observed in the number of CD cases over the period of 2003–2012 (Figure 1). The median age at diagnosis was 33.6 (interquartile range, IQR: 40.0–27.9) years. As shown in Figure 2, the age-specific frequency analysis showed that the peak incidence of CD was in the 21–30 years age group.

### 3.2. Disease Classification

For adult patients, the most common disease location was the ileo-colon (39.2%), followed by colonic (34.0%) and isolated ileal (26.8%) disease. As for disease behavior, 59.5% patients were identified to be inflammatory, followed by stricturing (32.5%) and penetrating (8.0%), as shown in Table 1.

For pediatric patients, the most common disease location was ileo-colon (50.0%), followed by distal 1/3 ileum (26.9%) and colonic (23.1%). As for disease behavior, 42.3% were identified to be inflammatory disease, followed by stricturing (34.6%), penetrating (19.2%), and both penetrating and stricturing (3.8%), as shown in Table 2.

### 3.3. Incidence, Disease Map, and Correlation with Affluence

**Figure 3:**
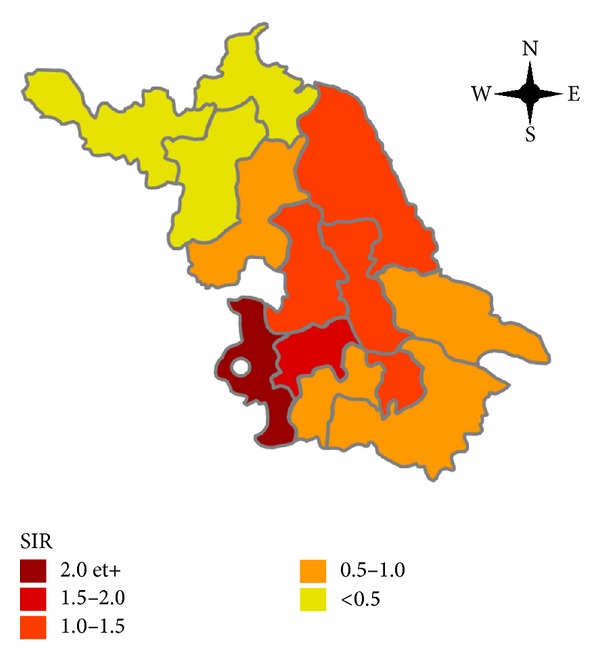
Geographic variations of SIRs of CD in Jiangsu province. Jinling Hospital has been marked out as a white dot.

The incidence rates of CD ranges from 1.3 cases/10^6^ population in the north area to 21.4 cases/10^6^ population in the south area. Age-sex specific incidence was provided in Table 3. According to the SIR of different provinces, the disease map of CD showed a marked geographic variability across the Jiangsu province (Figure 3). We found that the south region of Jiangsu province showed higher incidence of CD compared with that of north. The location of Jinling Hospital was also marked out in Figure 3. Regionally, the SIR of CD ranges from 0.15 in the north area (Xuzhou) to 2.42 in the south area (Nanjing).

We further investigated the relationship between economy status and CD incidence. Spearman correlation analysis showed a positive correlation between local CD incidence and average disposable expenditure (rh = 0.637; *P* = 0.019), living expenditure (rh = 0.659; *P* = 0.014), and GDP per capita (rh = 0.648; *P* = 0.016). However, there is no significant correlation between CD incidence and average saving deposits, annual income, or household Engel coefficient (Table 4).

## 4. Discussion

In the present study, we found that the number of CD cases increased steadily in Jiangsu province during 2003 to 2012. We also observed striking spatial variations in the distribution of CD in Jiangsu province. Map of SIR showed a pronounced geographic concentration of CD in the south area. Moreover, a positive correlation was observed between CD incidence and local economic status.

We do not know the exact reason of the increasing CD incidence in China. The speed of city industrialization in China is increasing in recent years, accompanied with surging economy and rising living standard. Although the economic growth was visible since the Reform and Open Policy, the gap between the poor and the rich is also increasing. We assumed that the increasing incidence of disease consistently observed as a society that becomes modernized or developed may be attributed to westernization of diet, changing lifestyle and antibiotic use, or improved hygiene status [16].

In the present study, the peak age for CD occurrence is 20–30 years and the age distribution of CD in current study is similar to reports from Western countries [17, 18]. Studies from Western countries showed that CD occurs 20%–30% more frequently in women [18, 19], while a male preponderance was observed in CD patients in the current study. We also realized that the proportion of isolated ileal disease in pediatric CD patients in our study is much higher than that of previous study [20]. We assume that this may be explained by the high frequency using of sigmoscope but not colonoscopy for the initial investigation at the department of pediatrics at our hospital, since parents believe that the examinations of sigmoscope and capsule endoscopy may be less harmful to their children than colonoscopy is.

The wide geographic variation of CD that we observed in the current study is consistent with other studies showing geographic variability in the incidence of CD [11, 21–25]. There are many possible reasons for the high degree of geographic variation. One of them is the increasing gap of economy status and living standards between different regions. In our study we observed a positive correlation between local CD incidence and average disposable expenditure (rh = 0.637; *P* = 0.019), living expenditure (rh = 0.659; *P* = 0.014), and GDP per capita (rh = 0.648; *P* = 0.016).

We do not know the exact mechanism of the association between the increasing CD incidence and rising economy status, but it may be explained by that people living in affluent areas spend more time in the office, in meetings, and at dinner, and they do less physical labor. Higher tension, more fast and fatty foods, and less physical exercise may be risk factors for CD, but these explanations need to be confirmed by further case-control studies in China.

Another possible explanation for the greater variability seen in current study is the study design, which is also a limitation of the current study. It is a single center study and our hospital is located in Jiangsu province. Hence, the geographic distribution of CD patients was influenced and limited by the location of our hospital and the distance between our hospital and different areas in Jiangsu province.

Finally, immigration of populations should also be considered as one of the aspects that may affect the geographic distribution of CD. Since the Reform and Open Policy in 1978, a large number of people have migrated to several affluent cities from rural or suburban areas to search for opportunities and a better quality of life. It is possible that such moves to urban zones have created new environmental pressures against which this population was not protected.

Previous studies have also shown an increased incidence of CD in affluent areas, classified using a social/material deprivation index [11, 24, 26, 27]. It has been suggested that higher incidence rates among those of higher socioeconomic status may be due to a delayed and/or low level of exposure to common infectious agents during childhood. This could be due to improved domestic hygiene, resulting in altered immune responses in genetically susceptible hosts, the so-called “hygiene hypothesis” [28].

The present study has several limitations. First, although the national medical insurance policy makes it easier to receive patients from all over mainland China and our hospital seems very attractive to CD patients from around China, especially Jiangsu province, it will still underestimate the true incidence and prevalence due to some underlying selection bias. A population-based multicenters study would be needed to explore the exact incidence and disease map of CD. Second, since the critical exposure factor in IBD is unknown, the lag time between exposure and disease onset is unknown, which raises a critical uncertainty in estimating incidence in relation to area of residence. It would be important to try to determine whether the critical area of residence is the one lived in at time of symptom onset (or within 2 years from diagnosis) or the residence of early childhood.

In conclusion, CD is an emerging disease in Jiangsu province. It affects predominantly young and middle-aged male patients. There is a marked geographic variability in CD incidence across Jiangsu province. CD incidence in affluent areas seems to be higher than that in less affluent areas. Further multicenter population-based studies are needed to assess the real disease map of CD.

## Figures and Tables

**Table 1:** Classification of adult Crohn's disease from Jiangsu province.

Variables	*n* = 627	Percentage (%)
Age at diagnosis		
A2 (17–40 y)	412	65.7
A3 (above 40 y)	215	34.3
Disease locations at diagnosis		
L1 (terminal ileal)	168	26.8
L2 (colonic)	213	34.0
L3 (ileocolonic)	246	39.2
L4 (Isolated upper disease)	22	3.5
Disease behavior at diagnosis		
B1 (inflammation)	373	59.5
B2 (stricturing)	204	32.5
B3 (penetrating)	50	8.0
P (perianal disease)	74	11.8

**Table 2:** Classification of paediatric Crohn's disease from Jiangsu province.

Variables	*n* = 26	Percentage (%)
Age at diagnosis		
A1a (0–10 y)	2	7.7
A1b (10–17 y)	24	92.3
Disease locations at diagnosis (%)		
L1 (distal 1/3 ileum)	7	26.9
L2 (colonic)	6	23.1
L3 (ileocolonic)	13	50.0
L4 (upper disease)	5	19.2
Disease behavior at diagnosis (%)		
B1 (inflammation)	11	42.3
B2 (stricturing)	9	34.6
B3 (penetrating)	5	19.2
B2B3 (penetrating and stricturing)	1	3.8
P (perianal disease)	4	15.4
Growth		
G0 (No evidence of growth delay)	6	23.1
G1 (growth delay)	20	76.9

**Table 3:** Age-sex specific incidence of Crohn's disease.

Gender by age group	Population (10^6^)	Number of cases	Incidence rate/10^6^
Males			
0–14	7.12	9	1.3
15–34	12.85	241	18.8
35–54	12.96	138	10.6
>55	8.01	33	4.1
Females			
0–14	6.04	3	0.5
15–34	12.43	97	7.8
35–54	12.58	96	7.6
>55	7.23	38	5.3
Total			
0–14	**13.16**	**12**	**1.8**
15–34	**25.28**	**338**	**26.6**
35–54	**25.54**	**234**	**18.2**
>55	**15.24**	**71**	**9.4**

**Table 4:** Correlation between CD incidence and local economic status in different areas of Jiangsu province.

Economic indicators	rh	*P *
Annual average saving deposits	0.341	0.255
Annual average income	0.445	0.128
Annual average disposable expenditure	0.637	**0.019**
Annual average living expenditure	0.659	**0.014**
GDP per capita	0.648	**0.016**
Household Engel coefficient	−0.146	0.635
